# Medical Jurisprudence and Toxicology

**Published:** 1836-04

**Authors:** 


### MEDICAL JURISPRUDENCE AND TOXICOLOGY.

*Experiments on Peroxide of the Hydrate of Iron as a Counter-poison to Arsenic. First Series.* By MM. MIGUEL and SOUBEIRAN, of Paris.

The French chemists have not been tardy in endeavouring to test the efficacy of this antidote to arsenic which was discovered by M. Bunzen. The following series of experiments were undertaken subsequently to those of M. Leseur, which were communicated to the Academy by Orfila, who considered that the German chemist had not exaggerated the importance of his antidote. The French experimenters found that if a large dose of arsenic was given to dogs and they were allowed to vomit, it produced no effect: it was necessary therefore to tie the œsophagus of those dogs on whom any experiments were to be made. As tying the œsophagus is a fatal operation, it was essential to ascertain the length of time that a dog would live after this alone was performed; and also the time which arsenic alone took to produce death when the œsophagus was tied after its introduction. A dog whose œsophagus was tied simply, died in seventy-eight hours: two other dogs, to whom nine and twelve grains of arsenic were given, and who were prevented from vomiting by tying the œsophagus, died in two hours and two hours and a half. In all the following experiments recently prepared peroxide of hydrated iron mixed with water was used, in the proportion of twelve parts to one of white arsenic.

Twelve grains of arsenious acid mixed with water was given to a little dog, and immediately afterwards the iron: the œsophagus was then tied; for some time he endeavoured to vomit, but two or three hours afterwards all symptoms had left: twenty-four hours after the operation the ligature was removed; fluids were given, but deglutition of solids was impossible, and the dog died on the sixth day. Two large dogs were similarly treated with a larger dose of arsenic (eighteen grains): the ligature was not removed. One lived seventy-eight, the other eighty-four hours.

The salutary effects of the hydrate of iron cannot be doubted in these three cases; for, from the previous experiment, the arsenic alone would have killed them in two or three hours, whereas they lived as long as they would have done had the ligature only been employed. Another series of experiments was performed to ascertain if the iron would arrest the effects of the poison after they had lasted some time. The poison was shown, by a previous experiment, to kill in two hours and a half: and this fact was confirmed by giving a dog eight grains of arsenic, after his œsophagus had been tied, and introducing the antidote two hours and a half afterwards: he died a quarter of an hour after this injection. To determine how long after the poison was taken the counter poison was useful, four experiments were made. Eighteen grains of arsenic were given to a middle-sized dog, the œsophagus was tied, and presently the efforts to vomit were very violent: an hour afterwards an opening into the œsophagus was made below the ligature, the iron was injected, and a second ligature applied. The animal lived ninety hours after the poison had been given. Two spaniels were treated in the same way, the antidote being injected one hour after the poison: eight grains of arsenic was given to one, who lived ninety hours; and twelve grains to the other, who lived thirty-eight hours. Eighteen grains were given to a large dog and the iron introduced *two* hours afterwards: he lived thirty hours.

In all four cases death was greatly delayed by the iron, and in the first experiment on a very strong dog the poison was neutralized, as he lived as long as if a ligature only had been applied. The conclusion must be admitted, that the iron acted as a counter-poison for some time after the arsenic had been swallowed. The unfavorable circumstances under which the antidote was given, strengthen this conclusion; the animals had undergone an operation which always produces on them great depression; the whole of the arsenic had been retained in the stomach for an hour or two, and nothing had been given to diminish its local or constitutional effects. In two experiments it was found that when arsenic was mixed with fatty matters, the iron was less efficacious as an antidote: probably from the arsenic being covered by the fat, and protected from the action of the iron.

The hydrated peroxide of iron used in these experiments was procured in the following way. Sulphate of iron of commerce was mixed with five or six times its weight of water in a platinum or porcelain capsule, and when it boiled, small quantities of nitric acid were added until the ruddy vapour ceased to ascend: this was to bring the oxide of iron to its maximum of oxygenation. The liquor was then diluted, and the iron precipitated by liquid ammonia. The precipitate was washed and mixed with a small quantity of water until it had the consistence of clear "bouillie." As it cannot be weighed in this state, thirty-six times as much sulphate of iron is required as the poison taken, for the sulphate contains one-third of its weight of peroxide. It should be kept as a hydrate, for the dried powder does not act as an antidote. The chemical change consists in a conversion of the arsenious acid into arsenite of iron. Three times the quantity of the hydrated peroxide of iron is sufficient to neutralize arsenious acid in solution, and the decomposition is instantaneous; but as the arsenic when used as a poison is almost always swallowed as a powder, a much larger proportion of the antidote is advisable, for the action goes on very slowly. Even then however it must neutralize all ill effects, if it is in contact with the arsenic, for as soon as the smallest quantity of the poison is dissolved, the oxide of iron acts upon it and precipitates it.

In conclusion it is recommended in all cases of poisoning by arsenic to give the patient large quantities of hydrate of the peroxide of iron; at the same time also to encourage vomiting, to get rid of undissolved arsenious acid from the stomach; and to repeat the hydrate as long as there are symptoms of any of the poison remaining.

*Bulletin Général de Thérapeutique, Decembre,* 1834.

---

*Second Series.* By Drs. G. Borelli and C. Demaria, of Turin.

Further experiments on this counter-poison to arsenic have been made in Italy, which are even more in favour of it than those performed by MM. Miguel and Soubeiran.

*Experiment* 1. Nine grains of arsenic were given to a dog, and immediately afterwards three ounces of the hydrated tritoxide of iron: the œsophagus was tied. Seven hours afterwards there were no symptoms of poisoning; it was found that the dog could swallow a little liquid; he could not vomit solids. The dog lived ten days; at that time he was killed, and it was found that the œsophagus had not been completely obliterated.

*Experiment* 2. A similar experiment was made on a dog with ten grains of arsenic; the ligature was removed after twenty-four hours: he lived twelve days; his deglutition was then free; he was killed by the same dose of arsenic given without the peroxide.

*Experiment* 3. A similar experiment, but the dog lived a fortnight.

*Experiment* 4. Fourteen grains of arsenic given to a large dog, and a ligature placed round the œsophagus, which was loosened half an hour afterwards to introduce an ounce of the peroxide. The dog wholly recovered, and served for another experiment five days afterwards.

*Experiment* 5. A similar experiment with six drachms of peroxide; the dog lived.

*Experiment* 6. Five drachms of peroxide introduced one hour after twelve grains of the poison, and the ligature finally removed in twenty-four hours: the dog died on the third day.

*Experiment* 7. Twelve grains of arsenic were given to a dog and the œsophagus was tied, but no peroxide given; death in three hours.

No free arsenious acid could be detected in the excrements of any of the animals. MM. Borelli and Demaria consider that four and a half parts by weight of the peroxide are required to neutralize one of arsenious acid. They conclude that it is as certain an antidote to arsenic as albumen is to corrosive sublimate. It should be kept as a soft mass from the contact of the air, lest it should absorb carbonic acid, which renders its combination with arsenic less easy. If it has absorbed carbonic acid, effervescence will be produced by the addition of muriatic acid. The injury to its efficacy by exposure was seen in one experiment where the peroxide had been prepared a fortnight previously, and, although it was given immediately after the poison, the dog died in twelve hours.

*Repertorio Medico-Chirurgico del Piemonte,* Marzo, 1835.

*On the Means of Preventing Poison.* By MM. Chevalier and Boys de Loury.

The attention of the authors of this essay was directed to this subject from the numerous trials on account of poisoning which took place in France; and they considered that an examination of the statistics of this crime might lead to measures of prevention. For this purpose they endeavoured to ascertain the number of the accused; the substances employed; the manner in which they were procured; the mode in which they were administered, &c. They collected the following particulars. During seven years 273 individuals have been accused of poisoning; 171 were acquitted and 102 condemned. Particular information could be procured regarding ninety-four of these cases only. More than half the number, that is fifty-four, were cases of poisoning with arsenic, seven with verdigris, five with corrosive sublimate, five with cantharides, four with nux vomica, three with fly-powder, two with nitric acid, one with opium, &c. As far as could be ascertained, the poison had been procured in many cases for the alleged purpose of poisoning vermin, and in others was used in the trade of the criminal. The causes of eighty-four of these crimes were, interest of twenty-eight cases, licentiousness of twenty-four, revenge of fifteen, jealousy of ten, and madness of six.

In eighty-one cases, the poison was administered thirty-four times in potage, two in milk, seven in flour, seven in wine, eight in bread, five in pastry, four in chocolate, four in medicine, two in coffee, one in cider, and two immediately by the mouth (to a woman when drunk, and to a child). Of ninety-four cases, sixty of the accused were men, and thirty-four were women. In many cases the taste or colour of the food with which the poison was mixed saved the victims from the danger to which they were exposed. Seven instances are related proving this. Such facts show that in many cases poisoning might be prevented by colouring, or rendering sapid those poisons whose action would not be injured by such a process. M. Berard has already proposed that arsenic should always be coloured, and the results of these researches justify such a step; for it appears that in sixty-two cases out of eighty-one the poison employed was white; that of these sixty-two there were fifty-four in which white arsenic was used, and that taste and colour in many instances warned the victims of their danger. On examining into the uses to which arsenic is applied it appears, 1. That almost all the white arsenic sold in the country districts of France is employed in the preparation of corn which is used as seed, and for the destruction of rats and mice. 2. That powdered metallic arsenic, or fly powder, is used to kill flies. 3. That white arsenic is used by veterinary surgeons in the diseases of animals, and by some persons for the itch. It does not appear that these uses of arsenic could be interfered with if it was coloured and rendered sapid, and therefore they propose, 1. That it would be useful and even indispensable to mix white arsenic employed for corn, and medicinally, with powdered aloes, in the proportion of ten parts of aloes to ninety of arsenious acid. 2. That arsenious acid employed to poison rats and mice should be mixed with Prussian blue, or soluble indigo in the proportion of ninety parts of arsenic to ten of colouring matter. 3. That "fly powder" should be mixed with a tenth part of its weight of soluble blue.

These plans would not only render poisoning less frequent, by increasing the difficulty of committing the crime, but would tend to prevent fatal accidents. Thus sixteen persons were poisoned at Bressières in 1828 owing to the same sack having been used for flour which had previously held the grain prepared by means of arsenic for seed: and eleven persons were injured by a similar accident at Bourbonne-les-Bains in 1833. *Journal de Chimie Médicale, Avril,* 1835.

[This is an ingenious application of the statistics of crime. Considerable difficulties exist in legislating in such matters; and, even if there were none, the correctness of the principle on which it is proposed to legislate is most questionable. Toffana, and the other secret Italian poisoners of the sixteenth century, carried on their horrid practices at a time when none of the virulent poisons were known which modern chemistry has discovered. The evil depends on a corrupt moral sense, which is beyond the power of any precautionary legislative acts. The colouring of arsenic might prevent accidents, but the wilful poisoner would select some other means.]

